# Successful Resection of Isolated Para-Aortic Lymph Node Recurrence from Advanced Sigmoid Colon Cancer following 156 Courses of FOLFIRI Regimen

**DOI:** 10.1155/2016/4548798

**Published:** 2016-08-25

**Authors:** Kaoru Takeshima, Kazuo Yamafuji, Atsunori Asami, Hideo Baba, Nobuhiko Okamoto, Hidena Takahashi, Chisato Takagi, Kiyoshi Kubochi

**Affiliations:** Department of Surgery, Saitama City Hospital, Saitama 336-0911, Japan

## Abstract

Isolated para-aortic lymph node (PLN) recurrence from colorectal cancer (CRC) is rare, with no currently validated treatments. Few reports have described the successful resection of isolated PLN involvement from CRC following chemotherapy. We report the case of a 63-year-old man who underwent sigmoidectomy for sigmoid colon cancer at our hospital. Pathological examination demonstrated advanced sigmoid colon cancer with metastatic involvement in both of the tested PLNs. Palliative chemotherapy was initiated four weeks after surgical resection, with administration of the FOLFIRI regimen. Four years after the operation, computed tomography (CT) revealed an enlarged PLN below the left renal vein. As PLN enlarged to 15 mm in the minor axis on a CT scan in 2014 after receiving a total of 156 courses of the FOLFIRI regimen, we considered the enlarged PLN to represent an isolated metastasis. Accordingly, lymph node resection was performed with microscopically negative margins. The patient maintained a good quality of life without any side effects throughout the whole course of his treatment and remains disease-free at 24 months without chemotherapy after resection of the isolated PLN. Curative resection following chemotherapy may improve survival of carefully selected advanced CRC patients with locoregional recurrence, such as isolated PLN involvement.

## 1. Introduction

Isolated para-aortic lymph node (PLN) recurrence of colorectal cancer (CRC) is rare and validated treatments have yet to be established; therefore, the prognosis of patients with isolated PLN recurrence is poor [1, 2]. Surgical resection for the local recurrence of colorectal cancer following chemotherapy is, in cases where complete resection is possible, the only modality shown to confer long-term survival [1, 3]. Salvage surgery for isolated PLN recurrence is likely to improve the survival of patients with metastatic CRC [1]. Palliative chemotherapy comprising two key drugs—irinotecan and oxaliplatin—combined with leucovorin (LV) and 5-fluorouracil (FU) for metastatic CRC has been shown to improve both progression-free survival and overall survival in patients with metastatic CRC [4]. Here we report the successful resection of isolated PLN recurrence in a patient who received a total of 156 courses of the FOLFIRI regimen following sigmoidectomy for sigmoid colon cancer.

## 2. Case Presentation

**Figure 1:**
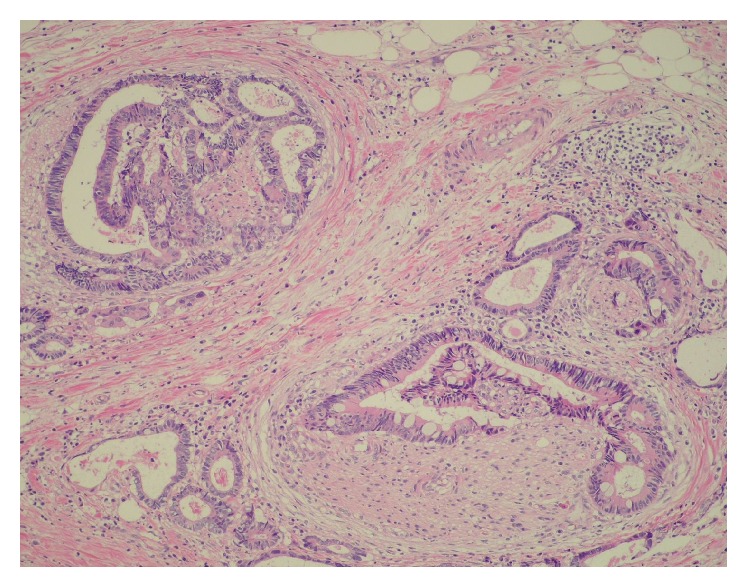
Histological examination of resected sigmoid colon cancer specimen. Histological examination revealed a well-differentiated adenocarcinoma. Hematoxylin and eosin stain (H&E), low magnification.

**Figure 2:**
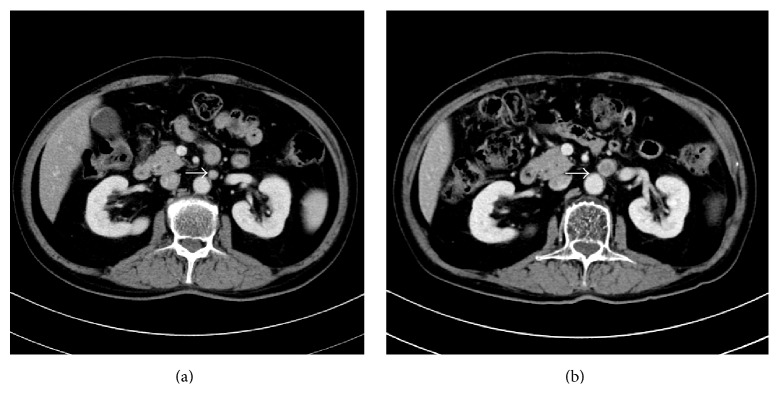
CT imaging of an enlarged para-aortic lymph node. Abdominal CT demonstrated an enlarged isolated para-aortic lymph node (a) in 2011. The lymph node was found to have enlarged to 15 mm in the minor axis (b) in 2014.

**Figure 3:**
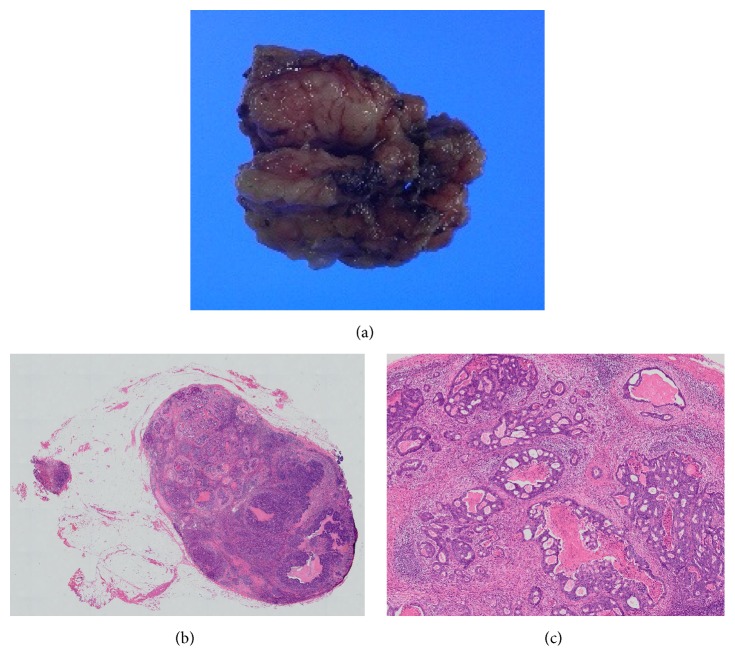
Histological examinations of the resected para-aortic lymph node. Histological examination revealed that the para-aortic lymph node was involved by well-differentiated adenocarcinoma with negative margins, with similar histology to the resected sigmoid colon cancer specimen. (a) Resected specimen, (b) H&E, original magnification, and (c) H&E, low magnification.

**Figure 4:**
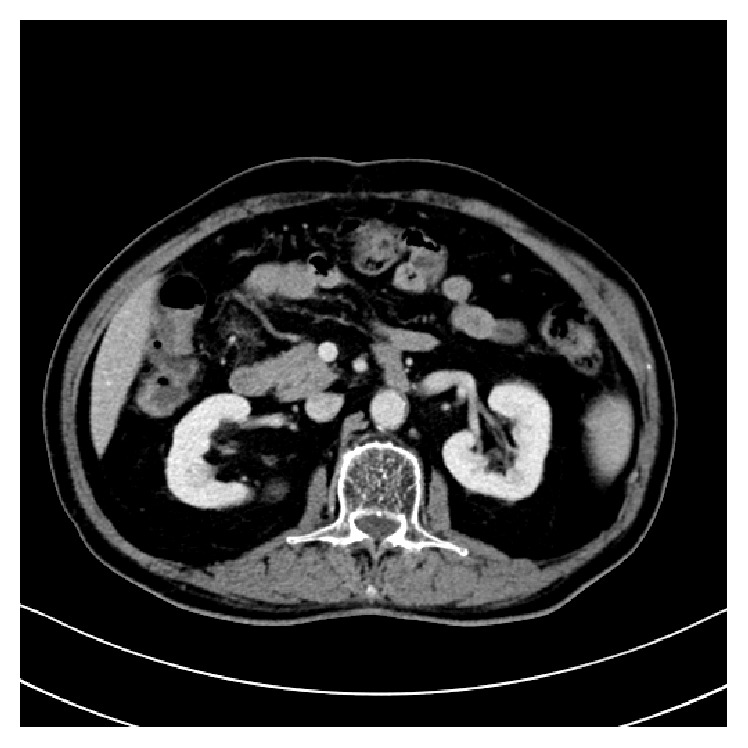
CT imaging performed in 2016. CT scan showed no evidence of para-aortic lymph node recurrence.

A 63-year-old man underwent sigmoidectomy for sigmoid colon cancer at our hospital in 2007. Histological examination of the surgical specimen revealed well-differentiated adenocarcinoma (Figure 1) that had invaded the subserosal layer (T3) with metastatic involvement in 12 of 20 dissected regional lymph nodes (N2b) and in both of the sampled PLNs (M1a). The tumor was retrospectively staged as IVA according to the TNM classification [5]. As residual disease was detected microscopically, palliative chemotherapy was initiated four weeks after surgical resection, with administration of the FOLFIRI regimen consisting of a 120-minute infusion of* l*-leucovorin 200 mg/m^2^ and a 90-minute infusion of irinotecan 150 mg/m^2^ followed by a 400 mg/m^2^ bolus of 5-FU and a 46-hour infusion of 5-FU 2400 mg/m^2^ every two weeks. Monoclonal agents were not combined for economic reasons. Computed tomography (CT) imaging was repeated every four months to assess the efficacy of chemotherapy. In 2011, following completion of the 92nd cycle of FOLFIRI, an abdominal CT scan demonstrated an enlarged PLN below the left renal vein (Figure 2(a)). Although we proposed positron emission tomography to diagnose whether the enlarged PLN was metastatic or not, he refused it. Under the informed consent of the patient, the FOLFIRI regimen has been continued since this time. The PLN was found to have enlarged to 15 mm in the minor axis on a CT scan performed in 2014 after the patient had completed a total of 156 courses of the FOLFIRI regimen (Figure 2(b)). The enlarged PLN was accordingly considered a metastatic at this time [6]. There was no elevation in his serum carcinoembryonic antigen levels throughout the course of the treatment. As there was no evidence of recurrence at any other sites on CT imaging; we elected to perform lymph node resection. Histological examination revealed involvement of the PLN by well-differentiated adenocarcinoma with negative margins, with similar histology to the resected sigmoid colon cancer (Figure 3). The patient did not suffer any adverse events, such as hematotoxicity or intestinal toxicity, at any time during the course of his treatment and has remained disease-free without chemotherapy for 24 months since resection of the isolated PLN, with no evidence of recurrence of sigmoid colon cancer (Figure 4).

## 3. Discussion

As the major sites of initial CRC metastases are the liver and lung; CRC metastases can commonly be resected. Curative resection of liver or lung metastases has been shown to improve survival in patients with metastatic CRC. Five-year survival rates of 30%–40% and 48% have been reported following the resection of liver and lung CRC metastases, respectively [7]. On the other hand, locoregional recurrence, which constitutes 10%–20% of all recurrences of CRC, is less common than recurrence at distant sites [8]; however, complete resection of locoregional recurrence has been shown to confer the same long-term survival in patients with liver or lung metastases [3]. Shibata et al. [9] and Bowne et al. [10] reported five-year median survival rates in patients with curative resection of locoregional recurrence from metastatic CRC of 81 months and 44 months, respectively. Moreover, the five-year survival rate of patients, who underwent curative resection of extraregional lymph node metastases including involvement of PLN from CRC, is significantly higher than that of patients who received palliative surgery (70.3% versus 12.5%) [11]. The incidence of isolated PLN recurrence is reportedly 1.3%, with a median survival time of 34 months in patients with resection of isolated PLN involvement and just 14 months in patients without resection [1]. Bae et al. [12] reported five-year survival rates of 33.9% in patients with curative resection of isolated PLN recurrence. These reports indicate that curative resection with microscopic negative margins of isolated PLN recurrence may improve the survival of patients with isolated PLN involvement. In the present case, salvage surgery with no residual tumor was likely to have contributed to the long-term survival of this patient.

Palliative chemotherapy comprising two key drugs—oxaliplatin and irinotecan—has demonstrated efficacy in improving the survival of patients with metastatic CRC [4]. Furthermore, the combination of monoclonal agents against angiogenesis or epidermal growth factor receptor (EGFR) has been shown to prolong overall survival better than that by chemotherapy alone [7]. In the present case report, the patient received FOLFIRI as a first-line regimen following sigmoid colon resection as a small bowel obstruction can develop due to peritoneal carcinomatosis from PLN involvement. A number of retrospective studies have reported that multidisciplinary therapy can improve the prognosis of patients with isolated PLN recurrence. Min et al. [1] reported a median survival time after recurrence of 14 months in 32 patients who received chemotherapy without resection of isolated PLN recurrence of whom 19 patients underwent radiation therapy plus concurrent or sequential chemotherapy. A median survival time of 37 months was reported in seven patients with isolated PLN recurrence from CRC who underwent stereotactic radiation therapy, with a median recurrence-free survival time of 26 months [2]. The major limitations of these reports are small sizes and retrospective nature of the studies. However, chemotherapy, radiation therapy, or chemoradiation therapy are likely to have efficacy in improving the overall survival time in patients with isolated PLN recurrence from CRC. Yasuda et al. [13] reported an interesting case of a patient with isolated PLN recurrence from CRC who has survived for 6 years with complete remission after chemoradiation therapy.

There are three major factors to consider regarding the treatment of cases such as the ones discussed in this study. First, there is a controversy regarding the use of immediate treatment or expectant management in patients with microscopic residual but asymptomatic disease following resection. A recent study reported that early treatment resulted in significantly prolonging the survival of asymptomatic patients with advanced CRC [14]. Conversely, a meta-analysis found no significant improvement in survival with early treatment of asymptomatic patients with advanced CRC [15]. In the present case, early treatment with FOLFIRI allowed successful resection of isolated PLN recurrence from CRC. Second, the use of intermittent or continuous immediate treatment remains contentious. No significant difference in the overall survival of patients with advanced CRC has been demonstrated between intermittent FOLFIRI and continuous FOLFIRI; however, the patients who underwent intermittent FOLFIRI are reportedly at a decreased risk of venous thromboembolism [16]. In the present case, FOLFIRI was immediately initiated and continued until 2014 when the patient underwent resection of an isolated PLN recurrence without any toxicities or adverse events. Third, the use of postoperative chemotherapy after the resection of PLN recurrence is controversial. Currently, as far as we know, there is no evidence that postoperative chemotherapy improves overall survival in patients with microscopically resected CRC recurrence [17]. In the present case, the patient has remained disease-free without chemotherapy for 24 months after resection of PLN recurrence.

Over a six-year period, our patient received a total of 156 continuous courses of the FOLFIRI regimen without any dose reduction or any pause and maintained a good quality of life with almost stable disease without any adverse events. To our knowledge, there have been no previous reports describing the long-term use of the FOLFIRI regimen as in our case, although a number of studies have reported that monoclonal antibody agents against angiogenesis or EGFR can be tolerated in long-term use [18, 19]. The long-term use of FOLFIRI was possible in our case for two reasons. First, it was indicated that, given the lack of adverse events during the course of the treatment, the patient's UGT1A haplotype contained neither UGT1A1^*∗*^6 nor UGT1A1^*∗*^28 [20]. Second, the cumulative toxicity, such as neurotoxicity from the cumulative use of FOLFOX, is rarely associated with that of FOLFIRI [4].

## 4. Conclusion

Here we report the successful resection of isolated PLN recurrence in a case of advanced sigmoid colon cancer following a total of 156 courses of the FOLFIRI regimen. Curative resection following chemotherapy may improve the survival of carefully selected advanced CRC patients with locoregional recurrence, such as isolated PLN involvement. The FOLFIRI regimen may be well tolerated in long-term use.
